# Multiple metabolic requirements for size homeostasis and initiation of division in *Saccharomyces cerevisiae*

**DOI:** 10.15698/mic2014.08.160

**Published:** 2014-08-01

**Authors:** Shivatheja Soma, Kailu Yang, Maria I. Morales, Michael Polymenis

**Affiliations:** 1 Department of Biochemistry and Biophysics, Texas A&M University, College Station, TX 77843, USA.

**Keywords:** START, elutriation, protein synthesis, growth rate, TDA1

## Abstract

Most cells must grow before they can divide, but it is not known how cells determine when they have grown enough so they can commit to a new round of cell division. Several parameters affect the timing of initiation of division: cell size at birth, the size cells have to reach when they commit to division, and how fast they reach that size. We report that *Saccharomyces cerevisiae* mutants in metabolic and biosynthetic pathways differ in these variables, controlling the timing of initiation of cell division in various ways. Some mutants affect the size at birth, size at initiation of division, the rate of increase in size, or any combination of the above. Furthermore, we show that adenylate kinase, encoded by *ADK1*, is a significant determinant of the efficiency of size control mechanisms. Finally, our data argue strongly that the cell size at division is not necessarily a function of the rate cells increase in size in the G1 phase of the cell cycle. Taken together, these findings reveal an unexpected diversity in the G1 cell cycle phenotypes of metabolic and biosynthetic mutants, suggesting that growth requirements for cell division are multiple, distinct and imposed throughout the G1 phase of the cell cycle.

## INTRODUCTION

In proliferating cells, the G1 phase of any given cell cycle lasts from the end of the previous mitosis until the beginning of DNA synthesis. In unfavorable growth conditions, *Saccharomyces cerevisiae* cells stay longer in G1, delaying initiation of DNA replication 1,2,3,4,5,6. Subsequent cell cycle transitions are less sensitive to growth limitations, and their timing does not vary greatly, even if growth conditions worsen. Thus, differences in the length of G1 account for most of the differences in total cell cycle, or generation times, between the same cells growing in different media 1,2,3,4,5,6. However, it is not clear how cells determine what growth requirements have to be met and how they are monitored so that cells can commit to a new round of cell division, at a point in late G1 called START. How nutrient, metabolic or other “growth” inputs activate the cell division machinery remains obscure. Historically, mutations in essential metabolic genes that arrest cell division at or before START have not received much attention. Such mutants were thought to resemble nutritionally limited cells because their growth in size was inhibited 6,7. Overall, it is not known if growth and metabolic requirements for cell division reflect hierarchical pathways, perhaps converging on a few specific biosynthetic needs. Alternatively, metabolic requirements for division may be multiple, distinct and imposed at different times from cell birth until commitment to a new round of cell division at START.

Decades ago, a relationship between the size or mass of a cell and the timing of initiation of DNA replication was shown from bacterial 8 to mammalian cells 9. A newborn budding yeast cell is smaller than its mother is, and it will not initiate cell division until it becomes bigger 1,2,6. These observations are consistent with the existence of a critical size threshold for initiation of division in yeast 10,11. How this critical size is set in response to metabolic cues, however, is unclear. It has been reported that the amount of G1 cyclins, which activate START, depends on both cell size and growth rate 12. Based on single-cell analyses, a recent report suggested that the rate of size increase in the G1 phase determines the critical size 13. In that scenario, slow growing cells would have a smaller critical size. Variations of G1 length among different mutants, or growth in different nutrients, could arise from differences in the size at which different mutants may enter and exit G1 and differences in the rate at which cells traverse G1. Measuring these variables (birth size, rate of size increase, critical size) in metabolic and biosynthetic mutants, and the extent to which any of these variables depends on one another is a necessary step towards deciphering the metabolic control of G1 progression and initiation of cell division.

Here, we identify nutritional requirements under which wild type cells adjust their critical size independently of the rate they increase in size in G1. We also show that cells lacking the kinase Tda1p specifically reduce their rate of size increase in response to different carbon sources, while their critical size remains unaffected, compared to wild type cells. Furthermore, from an analysis of mutants lacking enzymes of central metabolism or components of biosynthetic pathways, we identify several examples where birth size, rate of size increase, or critical size are affected independently of one another. Taken together, these results suggest that how cells set their critical size in not necessarily dependent on the rate cells increase in size in G1. Finally, the data we present are consistent with the notion that metabolic and biosynthetic requirements for division are multiple, distinct and imposed throughout G1, from cell birth until START.

## RESULTS

### Nutritional requirements and size homeostasis

**Figure 1:**
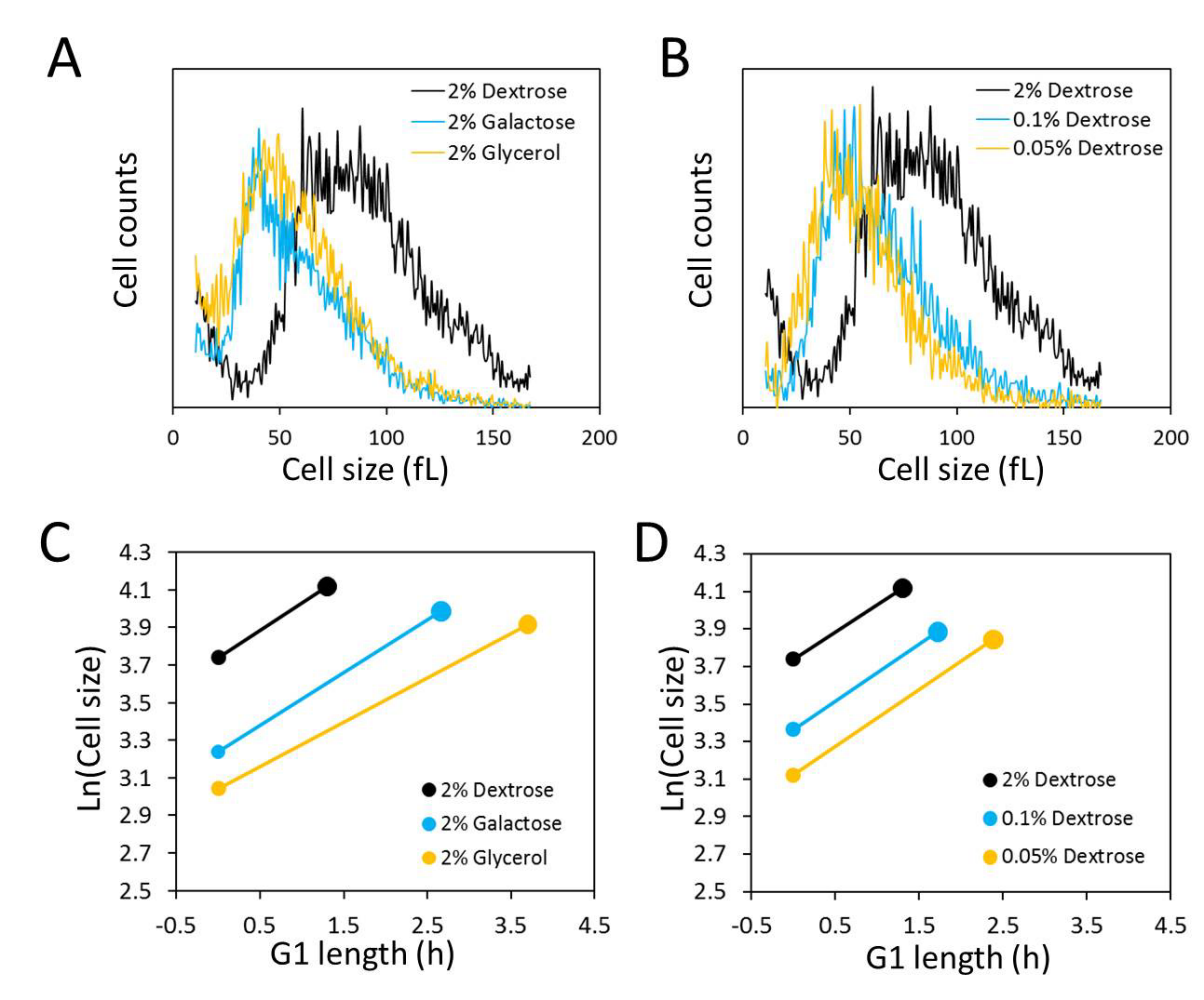
FIGURE 1. Nutrient control of size homeostasis and rate of size increase. **(A)** and **(B)** Cell size histograms of exponentially and asynchronously proliferating wild type diploid cells (strain BY4743), cultured in 1% w/v yeast extract, 2% w/v peptone and the indicated amount of the carbon source shown. The x-axis is cell size and on the y-axis is the number of cells. **(C) **and** (D) **Graphical representation of G1 variables in the growth conditions shown in A and B, using the values shown in Table 1. The x-axis is the calculated length of the G1 phase and the y-axis is the natural log of cell size at birth. The birth size at each condition is indicated with the smaller filled circle and the critical size with the larger filled circle. The length of the line connecting birth size with critical size is equal to the length of the G1 phase (T_G1_ in Table 1), and the slope of the line is equal to the specific rate of size increase (*k* in Table 1).

As described in numerous reports in the past (e.g., see 14), the poorer the carbon source in the medium used to culture *S. cerevisiae* cells, with galactose and glycerol being less favorable than glucose, the slower the population doubling time and the smaller the size of the cells. Recently, it was also reported that poorer carbon sources support a reduced rate of size increase in the G1 phase, causing a reduced size at the time of budding 13. As expected, compared to the cells grown in glucose (2% w/v), cell size distributions of asynchronous cultures of diploid BY4743 cells shifted to the left in galactose (2% w/v) or glycerol (2% w/v) media (Fig. 1A). From these experiments, we also calculated the daughter birth size (15, and Materials and Methods). Using galactose or glycerol as a carbon source led to a significant reduction in daughter birth size (Table 1), compared to the birth size of cells cultured with glucose. We also found similar trends toward a smaller population mean and daughter birth size in cells cultured with glucose as a carbon source but with the concentration of glucose dropping from 2% w/v to 0.1% w/v, or 0.05% w/v (Figure 1B and Table 1).

**Table 1:** G1 parameters in different nutrients of wild type and *tda1*∆/*tda1*∆ cells^a^. ^a^The strains were in the homozygous diploid BY4743 background. They were examined in at least 3 independent experiments, and in each experiment a technical duplicate was evaluated. The cells were cultured in 1% w/v yeast extract, 2% w/v peptone and the indicated amount of the carbon source shown in each case. ^b^Birth size was calculated from the size distributions of exponentially proliferating asynchronous populations, as described previously 15. The average of at least three independent measurements, with a technical duplicate for each measurement, and the associated standard deviation are shown in each case. ^c^The specific rate of size increase (k) and critical size were calculated from elutriated synchronous cultures as we described previously 17, assuming an exponential mode of growth. The average of at least three independent experiments and the associated standard deviation are shown in each case. ^d^These are G1 estimates from the formula: G1(hours)=Ln(Critical size/Birth size)/*k*. Note that these values reflect the G1 length of newborn daughter cells. For G1 length calculations, the errors (± sd) were not propagated.

**Strain**	**Medium**	**Birth size (fL)**	***k* (h^-1^)**	**Critical size (fL)**	**T_G1_ (h)**
*TDA1/TDA1*	2% Dextrose	42.1±1.0**^b^**	0.28±0.01^c^	61.5±0.6^c^	1.35^d^
*TDA1/TDA1*	2% Galactose	25.5±0.4	0.27±0.01	53.9±4.7	2.77
*TDA1/TDA1*	2% Glycerol	21.0±1.3	0.23±0.01	50.3±2.1	3.80
*TDA1/TDA1*	0.1% Dextrose	28.9±0.8	0.29±0.01	48.8±3.1	1.81
*TDA1/TDA1*	0.05% Dextrose	22.7±1.3	0.29±0.02	46.7±4.2	2.49
*tda1*∆*/tda1*∆	2% Dextrose	41.8±1.3	0.28±0.02	60.4±1.4	1.31
*tda1*∆*/tda1*∆	2% Galactose	26.3±0.9	0.24±0.01	52.6±0.7	2.89
*tda1*∆*/tda1*∆	2% Glycerol	21.8±0.6	0.19±0.02	52.7±2.9	4.65

To measure the rate of size increase and the critical size (defined here as the size at which half the cells in a synchronous population have budded) under all the above culture conditions, we then turned towards synchronous cultures obtained by centrifugal elutriation. An exponential mode of growth is thought to describe better the size increase of *S. cerevisiae* in G1 using single-cell photomicroscopy 10 or synchronous population monitoring by continuous volume measurements with a Coulter counter 16. Therefore, to calculate the rate of size increase, we incorporated the obtained values of cell size measured with a channelyzer into an exponential function. Cells proliferating in media with galactose and glycerol as a carbon source had a reduced rate of size increase compared to cells proliferating in glucose-containing medium (Fig. 1C and Table 1). In accordance with Ferrezuelo et al 13, there was a concomitant decrease (≈15-20%) in the critical size of cells in galactose and glycerol media, compared to cells in glucose medium (Fig. 1C and Table 1). Note that there was also a substantial decrease in the daughter birth size (≈75-100%) in cells proliferating in galactose or glycerol media (Figs. 1A, C and Table 1). Despite the reduced critical size, the smaller birth size and the reduced rate of size increase accounted for the much longer duration of the G1 phase in these carbon sources compared to growth in glucose (Fig. 1C and Table 1).

Similar experiments in media containing different concentrations of glucose revealed that limiting the concentration of glucose from 2% to 0.05% had no effect on the rate of size increase (Fig. 1D, and Table 1). Although the rate of size increase was unaffected, size homeostasis was altered significantly, to smaller daughter birth size (Fig. 1B and Table 1) and critical size (Fig. 1D and Table 1). The disproportionately greater reduction in birth size resulted in an increase in the length of the G1 phase in these cells (Fig. 1D and Table 1). Hence, at least within the range of glucose we used, these experiments provide an example where under physiological nutritional conditions critical size is set independently of the rate of size increase in G1.

### The kinase Tda1p contributes to the control of the rate of size increase in response to carbon source

**Figure 2:**
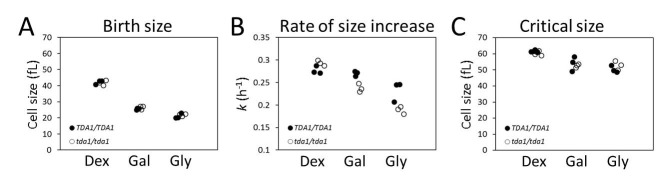
FIGURE 2: Loss of Tda1p reduces the rate of size increase but it does not affect the critical size or the birth size. **(A)** The birth size of *tda1*∆*/tda1*∆ cells and their *TDA1/TDA1* counterparts (in the BY4743 background) was measured from three independent experiments in each case, cultured in 1% w/v yeast extract, 2% w/v peptone and 2% w/v of either Dextrose (Dex), Galactose (Gal) or Glycerol (Gly). From synchronous, elutriated cultures (see Materials and Methods, and Table 1) of the same strains and media as in (A), we calculated the corresponding values for the specific rate of cell size increase constant *k* (in h^-1^) shown in **(B)**, and the critical size values shown in **(C)**.

To examine further the relationship between the rate of size increase and critical size, we focused on Tda1p because we had previously shown that cells lacking Tda1p had a prolonged G1 phase without altered size homeostasis 17. Here, we compared the birth size, rate of size increase and critical size of *TDA1/TDA1* vs. *tda1*∆*/tda1*∆ cells in cultures with glucose, galactose or glycerol as a carbon source. We found that while in all carbon sources the daughter birth size and critical size of *tda1*∆ cells were similar to the corresponding values of *TDA1^+^* cells (Figs. 2A, C), their growth decreased disproportionately in galactose and glycerol media (Fig. 2B). These results suggest that Tda1p plays a role in the mechanisms that determine growth rate in response to carbon source, and that the putative control of critical size by the rate of size increase is not evident in cells lacking Tda1p.

### Diverse G1 phenotypes of metabolic and biosynthetic mutants

**Figure 3:**
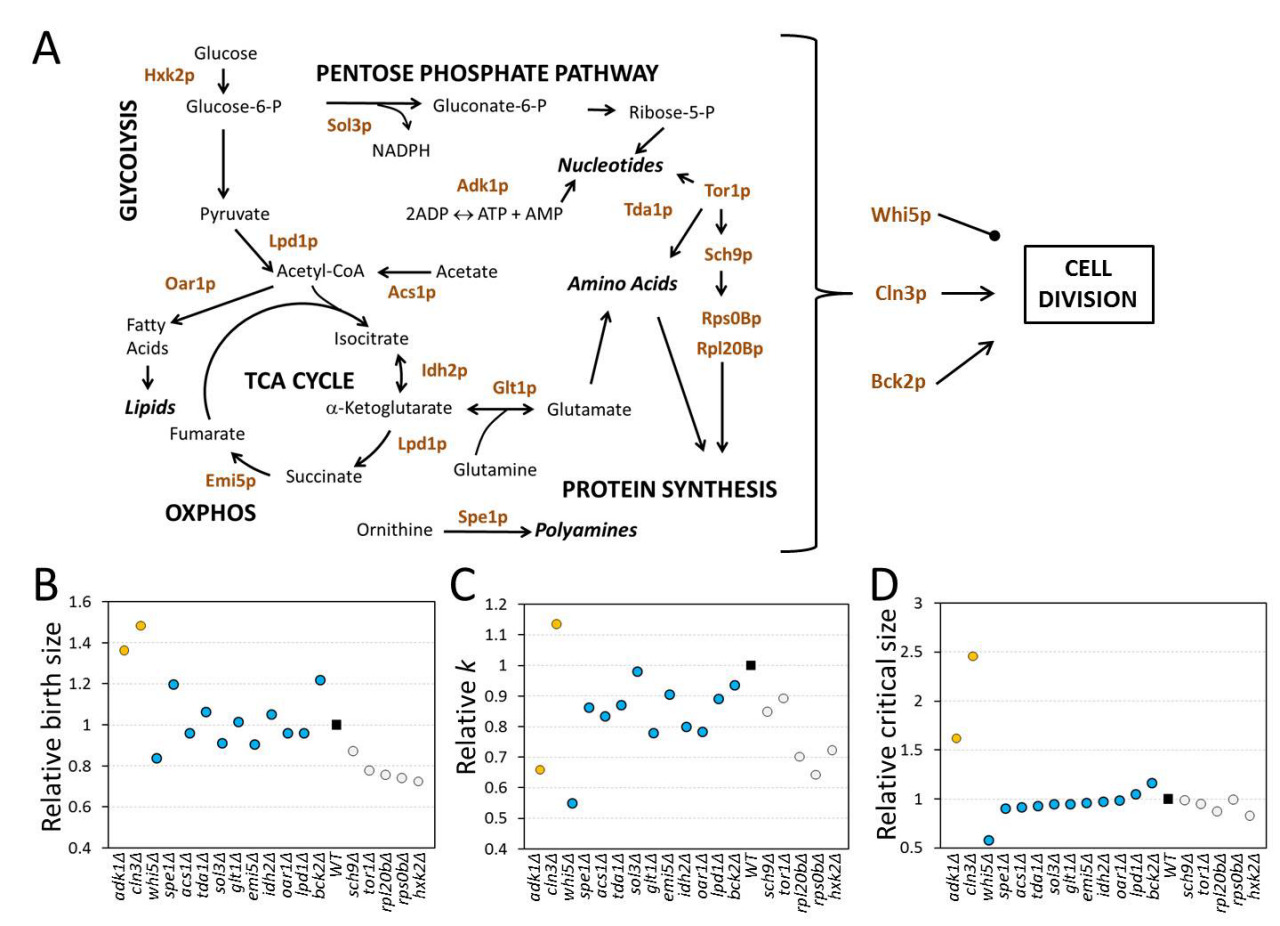
FIGURE 3: Diverse G1 phenotypes of metabolic and biosynthetic mutants. **(A)** Schematic overview of the reactions affected by the gene products we examined. This is a simplified view for clarity, missing numerous intervening reactions. **(B)** The birth size of each mutant shown on the x-axis was calculated for each deletion strain in the BY4741 and Y7092 background, shown in Table 2. For each gene deletion, the values from the two strain backgrounds were averaged, expressed relative to the corresponding value of the wild type, and shown on the y-axis. The gene deletions were group in three groups, based on principal component analysis and *k*-means clustering, using the R open source software, from the data shown in Table 2. The filled square is the wild type value. The relative specific rate of size increase **(C)** and critical size **(D)** are shown for each gene deletion, calculated and displayed as in (B), from the data shown in Table 2.

Next, to test if growth rate can be modulated independently of critical size, we reasoned that mutations that alter growth rate ought to be examined for their effects on critical size. Consequently, to further test the deterministic role of the rate of size increase on setting the critical size, we analyzed a set of 18 mutants, each lacking a single gene product functioning in diverse metabolic and biosynthetic pathways. We chose 10 single gene deletions that impair different reactions of central metabolism (Fig. 3A). We also analyzed strains lacking the Rps0Bp and Rpl20Bp ribosomal proteins and three kinases, including Tda1p (Fig. 3A). Tor1p has a general, well-described pro-anabolic role 18, while Sch9p, the yeast S6 kinase ortholog, regulates ribosome biogenesis downstream of Tor1p 18. Growth pathways must ultimately activate the cell division machinery. The components of the cell division machinery we included here are thought to function in the earliest steps of the switch that triggers cell division. Cln3p is an activating cyclin subunit of the major Cdk in yeast, Cdc28p (Cdk1p) 19. Bck2p is a protein that functions in parallel with Cln3p to activate transcription of cell cycle genes 20. Cells lacking both Cln3p and Bck2p are not viable 21. Whi5p is a repressor of the late G1 transcriptional program. Commitment to division is marked molecularly by nuclear eviction of Whi5p 11. We examined each gene deletion in the standard BY4741 background, using commercially available deletion strains 22. To better interpret the obtained results and minimize artifacts due to suppressors accumulated during or after construction of these haploid strains in the BY4741 background, we independently constructed the same gene deletions in the Y7092 background (23, see Materials and Methods). Together these two sets of mutants also enable construction of any desired double mutant combination in future experiments. The genotype of all the deletion strains in both backgrounds was verified, and we then measured their birth size, rate of size increase, and critical size in standard YPD medium with 2% w/v dextrose (see Table 2).

**Table 2:** G1 parameters of "growth" and cell cycle mutants^a^. ^a^The strains were examined in at least one experiment in each background, and in each case a technical duplicate was evaluated. The cells were cultured in 1% w/v yeast extract, 2% w/v peptone, 2% w/v Dextrose. All the parameters were calculated as described in Table 1 and in the Materials and Methods section.

**ORF**	**Strain**	**Birth size (fL)**	***k* (h^-1^)**	**Critical size (fL)**	**T_G1_ (h)**
NA	BY4741	21.9	0.35	41.2	1.81
*ACS1*	*acs1*∆*::KanMX*	20.2	0.3	39.1	2.17
*ADK1*	*adk1*∆*::KanMX*	33	0.25	66.6	2.82
*BCK2*	*bck2*∆*::KanMX*	28.3	0.35	49.9	1.64
*CLN3*	*cln3*∆*::KanMX*	33.6	0.41	120	3.14
*EMI5*	*emi5*∆*::KanMX*	18.8	0.29	40	2.62
*GLT1*	*glt1*∆*::KanMX*	21.4	0.27	40.2	2.32
*HXK2*	*hxk2*∆*::KanMX*	15.6	0.3	37.4	2.88
*IDH2*	*idh2*∆*::KanMX*	24.1	0.27	40.1	1.92
*LPD1*	*lpd1*∆*::KanMX*	21.1	0.33	46.1	2.34
*OAR1*	*oar1*∆*::KanMX*	21.6	0.3	44.9	2.47
*RPL20B*	*rpl20b*∆*::KanMX*	16.7	0.26	37.1	3.08
*RPS0B*	*rps0b*∆*::KanMX*	15	0.25	44.5	4.37
*SCH9*	*sch9*∆*::KanMX*	17	0.29	44.2	3.25
*SOL3*	*sol3*∆*::KanMX*	22.4	0.3	40.8	1.98
*SPE1*	*spe1*∆*::KanMX*	25.8	0.26	36.9	1.37
*TDA1*	*tda1*∆*::KanMX*	24.4	0.33	42	1.64
*TOR1*	*tor1*∆*::KanMX*	17.9	0.35	42.3	2.47
*WHI5*	*whi5*∆*::KanMX*	17.3	0.19	25.5	2.04
NA	Y7092	19.5	0.35	46.9	2.53
*ACS1*	*acs1*∆*::NatMX*	19.4	0.28	41.1	2.71
*ADK1*	*adk1*∆*::NatMX*	23.7	0.21	76.3	5.54
*BCK2*	*bck2*∆*::NatMX*	22.4	0.31	52.3	2.79
*CLN3*	*cln3*∆*::NatMX*	27.9	0.39	94.1	3.14
*EMI5*	*emi5*∆*::NatMX*	18.5	0.34	44.5	2.56
*GLT1*	*glt1*∆*::NatMX*	20.4	0.27	43.3	2.77
*HXK2*	*hxk2*∆*::NatMX*	14.3	0.2	35.2	4.48
*IDH2*	*idh2*∆*::NatMX*	19.4	0.29	45.4	2.91
*LPD1*	*lpd1*∆*::NatMX*	18.6	0.29	46.1	3.16
*OAR1*	*oar1*∆*::NatMX*	18.2	0.25	41.5	3.31
*RPL20B*	*rpl20b*∆*::NatMX*	14.6	0.23	39.8	4.35
*RPS0B*	*rps0b*∆*::NatMX*	15.5	0.2	42.7	5.08
*SCH9*	*sch9*∆*::NatMX*	18.9	0.3	42.5	2.73
*SOL3*	*sol3*∆*::NatMX*	15.5	0.38	42.4	2.65
*SPE1*	*spe1*∆*::NatMX*	23.7	0.34	42.8	1.73
*TDA1*	*tda1*∆*::NatMX*	19.7	0.28	39.3	2.5
*TOR1*	*tor1*∆*::NatMX*	14.3	0.28	40.8	3.81
*WHI5*	*whi5*∆*::NatMX*	17.3	0.19	25.3	2

Most mutants had an increased length of the G1 phase, consistent with the notion that growth and metabolism are required for initiation of cell division. Surprisingly, cells lacking ornithine decarboxylase, Spe1p, which catalyzes the first step in polyamine biosynthesis 24, had a shortened G1 phase compared to wild type cells in both strain backgrounds (Table 2 and Fig. 3). The rate of size increase of *spe1*∆ cells was lower than the corresponding value of wild type cells (Table 2 and Fig. 3C). However, this effect was countered by the larger birth size (Table 2 and Fig. 3B) and slightly smaller critical size (Table 2 and Fig. 3D) of *spe1*∆ cells, shortening the overall length of the G1 phase. The only other case with a reduced length of the G1 phase was *whi5*∆ cells (see Table 2), which was expected given the well-established role of Whi5p as a repressor of START 11. In agreement with previous results 25, the rate of size increase of *whi5*∆ cells was significantly reduced (Table 2 and Fig. 3C), but the shortened G1 of these cells is due to their greatly diminished critical size (Table 2 and Fig. 3D).

Our data suggest that although size homeostasis was affected in *bck2*∆ cells, displaying larger birth and critical sizes, the net effect in the duration of the G1 phase was minimal (Table 2). In the BY4741 background, the large birth size of *bck2*∆ cells was more pronounced, leading even to an apparent shortening of the G1 phase in that strain background (Table 2). Because cells lacking both Bck2p and Cln3p are not viable, Bck2p was thought to have a significant role at START, in parallel to Cln3p 21. However, later work showed that Bck2p has a rather generic transcriptional role in early G1 20, perhaps explaining the data we present here.

As mentioned earlier, the remaining mutants had a longer G1 phase. However, the mutants varied in their behavior not only quantitatively, but also qualitatively, due to different combinations of variables in each case accounting for the lengthening of the G1 phase (see Table 2 and Fig. 3). To determine if the single mutants represent distinct classes, we used principal component analysis. We found that three principal components accounted for >90% of the observed variance. We then used *k*-means clustering to assign the single mutants into the three clusters shown with different colors in Figs. 3B-D. Due to their very large birth and critical size, *adk1*∆ cells lacking adenylate kinase and *cln3*∆ cells were clustered together and separately from all other mutants (Fig. 3). The increase in the critical size we observed for *cln3*∆ cells was very significant, especially in the BY4741 background (Table 2). The value we obtained for *cln3*∆ cells (120 fL) was the average of two independent experiments (yielding 114 fL and 126 fL), and it was nearly three-fold higher than that of wild type cells (41.2 fL). In the Y7092 background the critical size of *cln3*∆ cells was still quite large (94 fL), two-fold greater than that of *CLN3^+^* Y7092 cells (46.9 fL; see Table 2). Hence, on average, loss of *CLN3* leads to a 2.5-fold larger critical size than wild type cells. Earlier elutriation experiments done in the W303 background (which is different from the S288c ancestry of the BY4741 and Y7092 strains) by the Nasmyth group (26; see Fig. 3 of that paper) also revealed that in YP Raffinose medium *cln3*∆ cells had to grow in size by more than 2.5-fold to reach the same budding index as wild type cells. It should be noted that the overall size increase in *cln3*∆ cells is to a significant extent due to enlargement of the vacuolar compartment 27,28. The critical size enlargement of *adk1*∆ cells (60-70% in both the BY4741 and Y7092 strains; see Table 2) was substantial but not as dramatic as that of *cln3*∆ cells. Interestingly, however, despite their very large critical size, *adk1*∆ and *cln3*∆ cells displayed opposite trends in their rate of size increase. Compared to wild type cells, *adk1*∆ and *cln3*∆ cells had reduced vs. increased growth rate, respectively. Hence, this is one more example where the rate of size increase does not apparently determine the critical size.

Consistent with the known roles of the Tor1p and Sch9p kinases in ribosome biogenesis and protein synthesis 18, *tor1*∆ and *sch9*∆ cells clustered together with *rps0b*∆ and *rpl20b*∆ cells, characterized mostly by a significant reduction in both their birth size, and their rate of size increase (Fig. 3). Interestingly, cells lacking the major yeast hexokinase 29, Hxk2p, also clustered into the same group (shown in light gray in Figs. 3B-D). As in the previous examples we discussed, a drop in the rate of size increase was not necessarily correlated with a reduction in critical size (e.g., in *sch9*∆ cells and *rps0b*∆ cells, see Figs. 3C, D). Although chemically inhibiting the catalytic activity of Sch9p has been reported to decrease critical size 30, we found that a complete deletion of *SCH9* does not (see Table 2). Instead, *sch9*∆ cells are born small, explaining their small overall size phenotype reported previously 31, and *sch9*∆ cells also grow in size slower (30, and Table 2). These phenotypes account for the long G1 of *sch9*∆ cells reported when *SCH9* was first identified (32, and Table 2).

The remaining mutants all apparently clustered in the same group (shown in blue in Fig. 3). There was no clear and consistent relationship among the variables we examined. For example, *spe1*∆ cells were born large, had a reduced rate of size increase and yet their critical size was slightly smaller than normal (Table 2 and Fig. 3). Overall, at least within the set of mutants we examined, we did not observe a significant deterministic relationship between the rate of size increase and critical size. Furthermore, the behavior of metabolic mutants is not uniform at all, arguing for distinct and diverse growth requirements in the G1 phase of the cell cycle.

### Adenylate kinase, Adk1p, has a major role in the efficiency of size control mechanisms

**Figure 4:**
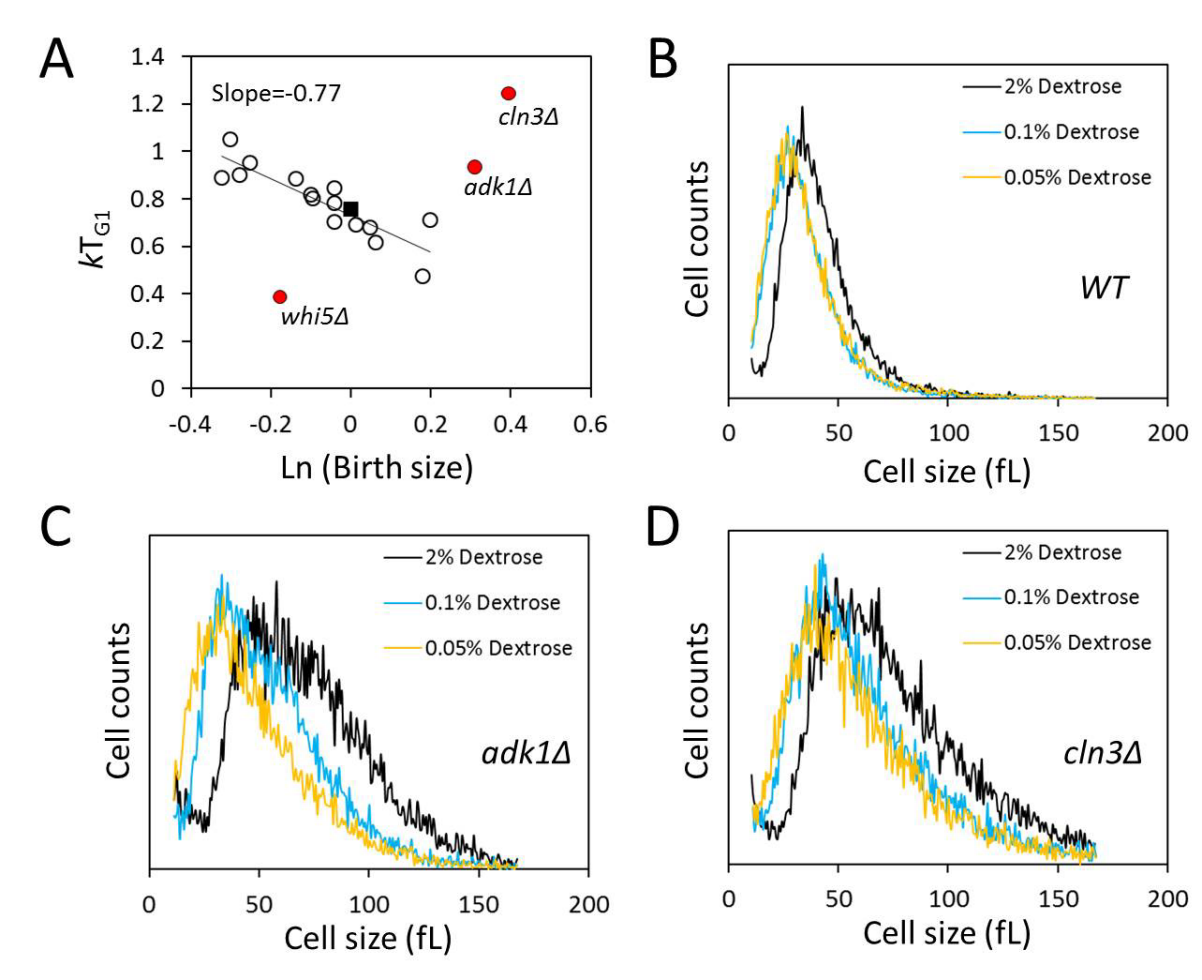
FIGURE 4: Adenylate kinase has a role in the efficiency of size control, but cells lacking Adk1p still adjust their size in response to nutrients. **(A)** In most mutants we examined (shown with open circles) size control operates efficiently. The filled square is the wild type value. On the x-axis is the natural logarithm of the normalized birth size values used in Fig. 3 (with the wild type values equal to one), which were obtained from the data in Table 2. These were plotted against their relative growth in size during the G1 phase (*k*T_G1_, y-axis). The values we used were the average from the two strain backgrounds. The line is a linear fit obtained with the regression function of Microsoft Excel, from all the strains except those shown in red. **(B-D)** Cell size histograms of exponentially and asynchronously proliferating wild type haploid cells of the indicated genotype (all in the Y7092 background) cultured in 1% w/v yeast extract, 2% w/v peptone and the indicated amount of the carbon source shown. The x-axis is cell size and the y-axis is the number of cells.

We next asked about the efficiency of size control mechanisms in the mutants we analyzed. Most of the mutants we queried had altered birth size, rate of size increase or critical size, and altered kinetics of G1 transit (see Fig. 3 and Table 2). However, despite altered size homeostasis and growth rate in many of these mutants, the question is whether such mutants still maintain the mechanisms that enable them to grow enough in size in G1, at a level comparable to that of wild type cells, before initiating a new round of cell division. Plotting the logarithm of birth size against the relative growth in the G1 phase of the cell cycle displays the efficiency of cell size control mechanisms 10,11. In such plots, a slope of zero indicates no size control. Based on photomicroscopy of single cells, wild type budding yeast daughter cells display a slope of -0.7 in such graphs, indicative of an imperfect but still significant size control 10,11. We applied this methodology to all the synchronous daughter cell populations of the strains we examined (Fig. 4A). In some cases we noticed differences between the BY4741 and Y7092 backgrounds, also between the two parental strains. For this reason, for the data we show in Fig. 4A, the values we used were the average from the two strain backgrounds. The strains shown in red were clear outliers from the rest (Fig. 4A). This was not surprising for *cln3*∆ and *whi5*∆ cells, which represent known and prototypical examples of inefficient size control 10,11. Cells lacking Whi5p do not wait long enough, while cells lacking Cln3p wait too long, before initiating a new round of cell division, respectively. Interestingly, we found that cells lacking Adk1p also had very inefficient size control (Fig. 4A). Adenylate kinase is a key metabolic enzyme, catalyzing the rapid return of the adenine nucleotide pool to equilibrium if the level of ATP, ADP or AMP is altered. To our knowledge, this is the first time that a significant role for adenylate kinase in the efficiency of size control has been described, in any system. The remaining strains, however, displayed efficient size control, with an overall linear fit of a slope of -0.77 (Fig. 4A). This included *bck2*∆ cells, suggesting that loss of Bck2p does not appear to significantly compromise the efficiency of size control, at least not to the same extent that loss of Cln3p, Whi5p or Adk1p does (Fig. 4A). We conclude that although most metabolic and growth mutants we examined have altered G1 variables, they nonetheless displayed cell size control that appeared to be as efficient as that of wild type cells.

Next, we asked if cells lacking Adk1p still respond to the nutrient control of cell size homeostasis, with cells getting smaller as the concentration of glucose is reduced (see Fig. 1B). At all conditions tested *adk1*∆ and *cln3*∆ cells remained massively larger than their wild type counterparts (Fig. 4). Nonetheless, the size of these cells was also progressively reduced as glucose levels were reduced (Fig. 4C, D). Therefore, despite the inefficiency of size control in *adk1*∆ and *cln3*∆ cells, the nutrient control of size homeostasis is largely independent of Adk1p and Cln3p.

## DISCUSSION

We discuss our results in the context of previous reports linking critical size with the rate of size increase, and we expand on the implications of our findings in regard with the cell cycle phenotypes of the mutants we examined.

Does the rate of size increase set the critical size for initiation of division? Although such a dependency may hold true in some cases, the following examples argue against that general rule proposed previously 13. First, in wild type cells we identified nutritional interventions that completely dissociate these two parameters: Reducing the glucose content of the medium drastically reduces birth size and critical size, but not the rate of size increase (Fig. 1). Second, even when nutrients simultaneously reduce both the rate of size increase, and the critical size, we identified contexts that one parameter is disproportionately affected. Cells lacking Tda1p reduce their critical size to the same extent as wild type cells do when cultured in media with poorer carbon sources (Fig. 2C). However, at the same time there was a disproportionate reduction in the rate of size increase of *tda1*∆ cells (Fig. 2B), which did not lead to an even greater reduction in critical size (Fig. 2C). Third, the correlation between the rate of size increase and critical size was not evident at all from our analysis of many mutants (Fig. 3 and Table 2). For example, *adk1*∆, *whi5*∆ and *rps0b*∆ cells all had a similarly compromised rate of growth, yet their critical sizes diverged widely, from very large (*adk1*∆ cells), to very small (*whi5*∆ cells) or slightly larger than normal (*rps0b*∆). Note also that even in cases with similarly dispersed population size distributions, the rates of cell size increase could diverge in opposite directions. For example, the cell size distributions of *cln3*∆ and *adk1*∆ populations were remarkably similar, displaying large variance. However, the relationship between the rate of size increase and critical size trended in the opposite direction in the two mutants. The rate of size increase was moderately increased in *cln3*∆ cells (Figure 3 and Table 2). In contrast, the rate of size increase was significantly reduced in* adk1*∆ cells (Figure 3 and Table 2). Finally, it was recently reported that several aneuploid strains display a reduced rate of size increase and a larger than normal critical size 33, providing yet another example of incongruence between the rate of size increase and critical size. Taken together, we think the physiological and genetic evidence we presented above argues against a general deterministic role of the rate of size increase in setting the critical size.

We think two major factors may account for the different conclusions we reached, compared to those of Ferrezuelo *et al.* 13, regarding the role of growth rate in setting the critical size. First, we examined a much broader array of nutritional and genetic interventions, including several gene products with distinct metabolic roles, under which the putative linkage between the rate at which cells increase in size and their critical size was clearly disrupted. Second, in accordance with previous reports 10,16, we calculated the increase in size based on an exponential mode, which incorporates size differences.

Our data reveal a multitude of ways that biosynthetic and metabolic mutants affect G1 progression. Among the mutants we examined, their birth size, rate of size increase and critical size were affected in virtually any combination. The most straightforward interpretation of these findings is that growth requirements for cell division do not reflect a single hierarchical pathway. Instead, it is more likely that growth requirements are multiple and that they are imposed throughout the G1 phase. Metabolic mutants that affect cell division have not attracted much attention in the past. Historically, several screens for regulators of initiation of cell division interrogated cell size 30,34,35,36,37. Using only critical size mutants to identify mechanisms that determine the timing of initiation of cell division obviously does not allow the sampling of gene products that do not affect critical size. A prime such example is cells lacking Tor1p. The key growth signaling role of Tor1p is well established 18, yet the critical size of *tor1*∆ cells is normal (Fig. 3). The phenotype of other mutants is even more subtle. For example, loss of Tda1p affects neither the birth size nor the critical size, only the rate of size increase and that only on poor carbon sources (Fig. 2). Tda1p is a kinase of unknown function, originally identified as a modifier of topoisomerase I-induced DNA damage 37. Interestingly, the human ortholog of Tda1p, NUAK1 (based on predictions by the P-POD program at http://ppod.princeton.edu ), is an AMPK-related protein kinase, with roles in metabolic homeostasis, tumorigenesis and senescence 39. Our findings regarding Tda1p’s role in different carbon sources in yeast are perhaps consistent with a conserved growth-related function of these kinases.

Most of the loss-of-function metabolic and biosynthetic mutants we examined had a prolonged G1 phase. Despite the delay in G1 progression, in most cases size control was still operational (Fig. 4A). In other words, although in these strains G1 progression was delayed due to altered size homeostasis, rate of growth, or both, these mutants still "knew" how much they had to grow in size before initiating a new round of cell division. Interestingly, this was not the case for cells lacking Adk1p (in addition to mutants lacking the well-known START regulators Cln3p and Whi5p, see Fig. 4A). Adk1p has a central role in maintaining the equilibrium in the concentration of ATP, ADP and AMP in the cell. The cellular energy charge, expressed as "half of the average number of anhydride-bound phosphate groups per adenine moiety" 40, is not altered by adenylate kinase. However, at any given value of energy charge, the actual proportions of ATP, ADP, and AMP, and the activity of any enzymes that respond to changes in those proportions are determined by adenylate kinase. Based on these considerations and the cell cycle phenotypes of *adk1*∆ cells we report, it is reasonable to speculate that Adk1p and possibly other proteins that respond to perturbations of nucleotide pools play a significant role in size control mechanisms.

In conclusion, with regard to when cells initiate division, our results suggest that "growth" mutants occupy a large and varied phenotypic space. Among the mutants we examined, there were numerous qualitative differences in G1 variables (see Table 2). The mechanistic basis of these differences is unclear at present and needs to await further experimentation. Nonetheless, a reasonable interpretation of these results is that metabolic and biosynthetic requirements for initiation of cell division are multiple and they are imposed throughout the G1 phase of the cell cycle. These growth requirements are likely the output of several metabolic pathways, acting perhaps in parallel. Defining the network arrangement of these metabolic outputs and how they impinge on the cell division machinery will illuminate the metabolic control of cell division.

## MATERIALS AND METHODS

### Strains and media

The strains we used are described in the corresponding Figures and Tables, and they were in the following backgrounds: BY4743 (MATa/α *his3*Δ*1*/*his3*Δ*1 leu2*Δ*0*/*leu2*Δ*0 lys2*Δ*0*/*LYS2 MET15*/*met15*Δ*0 ura3*Δ*0*/*ura3*Δ*0*); BY4741 (MATa *his3*Δ*1 leu2*Δ*0 met15*Δ*0 ura3*Δ*0*); Y7092 (MAT( *can1*∆*::STE2pr-*Sp*_his5 lyp1*Δ* ura3*Δ*0 leu2*Δ*0 his3*Δ*1 met15*∆*0*) - a gift from Dr. C. Boone (Univ. of Toronto). Single gene deletion mutants in the BY4741 background were generated by the Yeast Deletion Project 22. The corresponding deletions in the Y7092 background were constructed exactly as described previously 23. The genotype of all strains was verified by PCR, for the presence of the replacement cassette and the absence of the corresponding ORF in each case.

### Cell size measurements

All size measurements were performed with a Z2 Beckman Coulter Channelyzer. In experiments where the population mean was recorded, we measured the geometric mean of the cell size distribution of the population, using the AccuComp software package that accompanies the instrument. For each sample, we evaluated two cell dilutions, differing two-fold in the concentration of cells. The average of these two measurements was recorder for a single experiment. For birth size measurements of asynchronous, exponentially proliferating cells, we focused on the left of mode area of the distribution. From that area of the histogram we recorded the largest value of the 10% smallest cells, as we have described previously 15. In all cases where we report either a birth size value or a population mean for any given strain and condition, we report the average of at least three independent experiments, each performed as we described above.

### Elutriations

We used a J6-ME Beckman centrifugal elutriator to obtain highly synchronous, early G1 cells. We have described in detail elsewhere the methodology for elutriation experiments 17. Briefly, for each experiment, we loaded a 250 ml culture in late exponential phase (for YPD cultures, the cell density was 2-5E+7 cells/ml) and collected the early G1 cell suspension at 2,400 rpm centrifugal speed and 40 ml/min, or 50 ml/min, pump speed for haploid, or diploid strains, respectively. The percentage of budded cells was typically 0-1%, and rarely exceeded 5%, with the exception of *whi5*∆ cells, which were typically 10-15% budded. The cell density was adjusted to about 1E+7 cells/ml. Every 20 min afterwards, aliquots were taken to measure the fraction of budded cells with a phase microscope and the cell size of the population as we described above. From these data, we plotted the natural logarithm of the cell size (y-axis) as a function of time (in hours, on the x-axis). From the slope of these graphs, we obtained the specific rate of size increase, *k*. We also plotted the fraction of budded cells (y-axis) as a function of cell size (in fL, on the x-axis). After the fraction of budded cells began to increase, we fitted the linear portion of these graphs to a straight line using the regression function of Microsoft Excel, and calculated the critical size for 50% of budded cells. To estimate the length of the G1 phase (T_G1_), we used the exponential growth equation Ln(Critical size/Birth size)=*k*T_G1_ using the values of the corresponding variables calculated as we described above.
